# Hormonal Contraceptive Use During Relationship Formation and Sexual Desire During Pregnancy

**DOI:** 10.1007/s10508-015-0662-6

**Published:** 2015-12-24

**Authors:** Kelly D. Cobey, Jan Havlíček, Kateřina Klapilová, S. Craig Roberts

**Affiliations:** 1 School of Natural Sciences, University of Stirling, Stirling, Scotland, UK; 2 Faculty of Science, Charles University, Prague, Czech Republic; 3 Faculty of Humanities, Charles University, Prague, Czech Republic; 4 Clinical Epidemiology Program, Ottawa Hospital Research Institute, Ottawa, ON K1H 8L6 Canada

**Keywords:** Congruency hypothesis, Pregnancy, Oral contraception, Desire, Menstrual cycle

## Abstract

Women who are regularly cycling exhibit different partner preferences than those who use hormonal contraception. Preliminary evidence appears to suggest that during pregnancy women’s partner preferences also diverge from those prevalent while regularly cycling. This is consistent with the general assertion that women’s mate preferences are impacted by hormonal variation. During pregnancy, women’s preferences are thought to closely resemble those displayed by women who are using hormonal contraception. Here, based on this literature, we compared levels of sexual desire among pregnant women who met their partner while using hormonal contraception and pregnant women who met their partner while regularly cycling. We predicted that women who met their partner while using hormonal contraception would experience higher levels of in-pair sexual desire during pregnancy since these women will have partner preferences that more closely match those prevalent at the time of their partner choice. Our results provided support for the idea that previous contraceptive use/non-use may impact subsequent sexual desire for the partner during pregnancy. Pregnant women who met their partner while using hormonal contraception (*N* = 37) were shown to have higher levels of in-pair sexual desire than those who met while regularly cycling (*N* = 47). In contrast, levels of extra-pair desire were not related to previous use/non-use of hormonal contraception. These findings were robust when controlling for a number of relevant individual difference variables known to impact sexual desire. Our results contribute to our understanding of factors affecting relationship functioning during pregnancy.

## Introduction

In the past decade, researchers have begun to consider what impact hormonal contraceptive use may have on women’s mating psychology. Hormonal contraceptive use reduces naturally occurring cyclical hormonal variance and, as a consequence, women who use hormonal contraception have been shown to display different mating preferences and behaviors than women who are regularly cycling (e.g., Alvergne & Lummaa, 2010; Jones et al., 2008; Penton-Voak et al., 1999). For example, work by Little, Burriss, Petrie, Jones, and Roberts (2013) has demonstrated that, upon initiation of hormonal contraception, women prefer lower levels of masculinity in their male partners. Moreover, these researchers showed that women who met their partner while using hormonal contraception were actually paired with men who had lower levels of masculinity as assessed via both facial measurements and perceptual judgments. This suggests that hormonal contraceptive use has the potential to influence women’s (adaptive) mate preferences (Alvergne & Lummaa, 2010; Roberts, Gosling, Carter, & Petrie, 2008).

Classic research on the impact of hormonal contraceptive use on women’s sexual desire has exclusively examined the impact of current use. Interestingly, these types of studies have produced a very mixed set of findings, with positive, negative, and null results all having been documented (reviewed in Burrows, Basha, & Goldstein, 2012; Pastor, Holla, & Chmel, 2013). To complicate matters further, it is often challenging to compare results from studies examining how women’s use of hormonal contraceptives impacts their sexual desire. For example, studies using samples of young women may be biased in that the initiation of hormonal contraception often coincides with the initiation of one’s first sexual relationship. It would seem inappropriate to compare such studies to the many studies that examine the impact of hormonal contraceptive use on sexual desire in married women. Moreover, comprehensive randomized-controlled studies which examine changes in sexual desire during use of various types and concentrations of hormonal contraception, and use a within-persons design, are lacking. The use of a within-persons design is particularly crucial since women who choose to use hormonal contraception could potentially differ on a number of dimensions from those who choose not to (e.g., socioeconomic status, education level, religious background, sociosexual orientation, relationship status, relationship length).

Given the importance of sexual satisfaction for relationship quality and overall well-being, lack of knowledge, and the mixed associations reported on this topic, are clearly dissatisfying. In light of these findings, we recently proposed the congruency hypothesis as a new framework for understanding how hormonal contraceptive use might impact women’s partner-specific sexual desire (Roberts, Cobey, Klapilová, & Havlíček, 2013, 2014a). We predicted that, because partner preferences differ when regularly cycling and when using hormonal contraception, a woman’s current level of sexual desire for her partner may be partially dependent on whether her current use/non-use of contraception is congruent with her use at the time at which she began her relationship. Thus, if a woman’s hormonal contraceptive use has changed since the time of partner choice, her mate preferences will be somewhat different, and therefore we predicted she may experience a moderate decrease in desire for her partner since he may no longer match her preferences as closely. However, we predicted that hormonal contraceptive congruency would not impact women’s sexual desire for extra-pair men since women can easily adjust the target of their extra-pair interest or fantasy. That is to say, if a woman’s mate preferences shift, she may continue to show consistent levels of general sexual desire to men other than her partner: she can easily find someone new in her environment who matches her new preferences. In contrast, since a woman’s partner is constant, if her mate preferences change, her sexual interest for him may change. To examine this possibility, it is necessary to distinguish the object of sexual desire (partner-focused vs extra-pair); a distinction that is rarely made in existing research (Roberts et al., 2014a).

Recent empirical research testing the congruency hypothesis has provided support for its premises. Roberts et al. (2014b) showed that women whose current contraceptive use was congruent with use at the start of the relationship had higher levels of sexual satisfaction in their relationship. Importantly, effects of congruency in this study did not extend to non-sexual aspects of the relationship. This finding supports earlier work by Roberts et al. (2012) who found that, among women who were currently regularly cycling, those who met their partner on the pill were less sexually satisfied and had lower levels of partner attraction than those who met while regularly cycling. Interestingly, however, these same women who met their partner on the pill were more satisfied with his provision of parental care than were those women who met their partner while regularly cycling. This finding may be explained by the fact that masculine traits, which are preferred at reduced levels when using hormonal contraception, tend to be associated with lower levels of paternal care (Boothroyd, Jones, Burt, & Perrett, 2007). These results appear to suggest that contraceptive use/non-use at the start of the relationship has implications for subsequent relationship satisfaction. In other work, we have also provided preliminary evidence that contraceptive congruency may impact other aspects of one’s relationship beyond sexual and romantic relationship satisfaction, including levels of sexual jealousy (Cobey, Roberts, & Buunk, 2013).

The current research was developed as an extension of the literature outlined above and in order to test the predictions of the congruency hypothesis. Here, we aimed to test how use/non-use of hormonal contraception at the time of relationship initiation is related to sexual desire levels during subsequent pregnancy. Pregnancy is a distinctive time for couples, and the combination of physical and emotional changes occurring may have consequences for both general and sexual aspects of the relationship. Previous research has demonstrated mixed results with respect to how pregnancy influences relationship quality and sexual satisfaction. For example, meta-analyses indicate that pregnant women exhibit substantial inter-individual variability, particularly in the second trimester, with respect to their sexual responsiveness, coital frequency, and sexual satisfaction (von Sydow, 1999). We suggest that the congruency hypothesis could potentially contribute toward explaining some of this variance.

Since hormonal changes across the cycle or as a result of hormonal contraceptive use impact women’s sexual desire and mating preferences, it is reasonable to presume that as a result of hormonal variation, pregnant women also experience changes in their social perception and thus in partner preferences. Pregnancy is maintained by hormonal changes; perhaps most notably, it is characterized by rapid changes in levels of estrogen and progesterone, which rise continuously from conception to parturition. At present, there is preliminary evidence to suggest that partner preferences when pregnant resemble those predominant during hormonal contraceptive use. For example, Jones et al. (2005) showed that women who were using hormonal contraceptives, or who were pregnant, have a stronger preference for healthy-looking faces than women who were regularly cycling. Thus, based on this literature, we hypothesized that a pregnant woman who met her partner while using hormonal contraception would have partner preferences that were more congruent than a pregnant woman who met her partner while regularly cycling. As a consequence, we expected that: (1) Women who met their partner while using hormonal contraception would display higher levels of sexual desire toward their partner during pregnancy than those who met their partner when regularly cycling; (2) In contrast, women’s sexual desire during pregnancy for extra-pair men would not relate to contraceptive congruency since women can adjust the target of their extra-pair interest.

## Method

### Participants

Participants were recruited via a month-long advertisement posted in the pregnancy section of a British social networking site for expectant mothers and entered into a draw to win a £40 gift card in exchange for their time. Specifically, women were told they were being invited to take part in a study about the psychological experience of pregnancy. We excluded women who indicated they were not pregnant, were presently single, non-heterosexual, or who indicated they were receiving hormonal treatment throughout their pregnancy (*N* = 23). Of the sample, 96.4 % indicated they were residing in the United Kingdom. Participants who clicked on our advertisement were directed to an online questionnaire where they were briefed about the study and subsequently provided informed consent. Participants were 84 pregnant women expecting a singleton birth. In total, 37 women indicated that they were using hormonal contraception at the time their relationship began, while 47 indicated they had been regularly cycling. Participants ranged in age from 19 to 43 years (*M* = 31.71, *SD* = 4.63), and women in the two groups did not differ significantly in age (*t* = 1.07, *p* = .29). Participants were between 7 and 40 weeks pregnant (*M* = 23.80, *SD* = 8.09), with the majority of women in their second trimester. Participants reported having between 0 and 5 existing children (*M* = .65, *SD* = .96). We also collected demographic information with respect to highest levels of education (five categories ranging from less than secondary school to postgraduate degree) and on relationship length (four groups; <1, 1 to <2, 2 to <4, 4 years or more; *M* = 3.52, *SD* = .90).

This research project received ethical approval from the University of Stirling Psychology Ethics Committee.

### Procedure

We first asked participants to complete some basic demographic questions and then to respond to some pregnancy specific questions (e.g., number of weeks pregnant, current use of fertility drugs or hormonal treatment). Participants were also asked to indicate whether they were using any form of hormonal contraception when they started their relationship with their current romantic partner. Specifically, we asked “When you first met your partner what type of hormonal contraception were you using?” with options none, combined oral contraceptive pill, progesterone only pill, and other hormonal contraceptive. We also confirmed that the participants’ current partner was the same person who fathered her pregnancy.

Finally, we assayed our participants’ sexual desire via two target specific items. Specifically, we asked participants: (1) In the past week, how much sexual desire did you feel toward your partner? (2) In the past week, how much sexual desire did you feel toward men other than your partner? Participants indicated their response to each of these items on a 100-point scale with endpoints being described as “much lower than average” and “much higher than average” and with higher scores denoting higher levels of desire. We used these two target specific questions rather than a general measure of sexual desire since failing to make this distinction potentially conflates two diverse types of sexual desire and experience (Roberts et al., 2013). The reference frame of the past week was used in order to minimize bias related to longer retrospective reporting and circumvent bias from specifying responses to a single day.

## Results

**Fig. 1:**
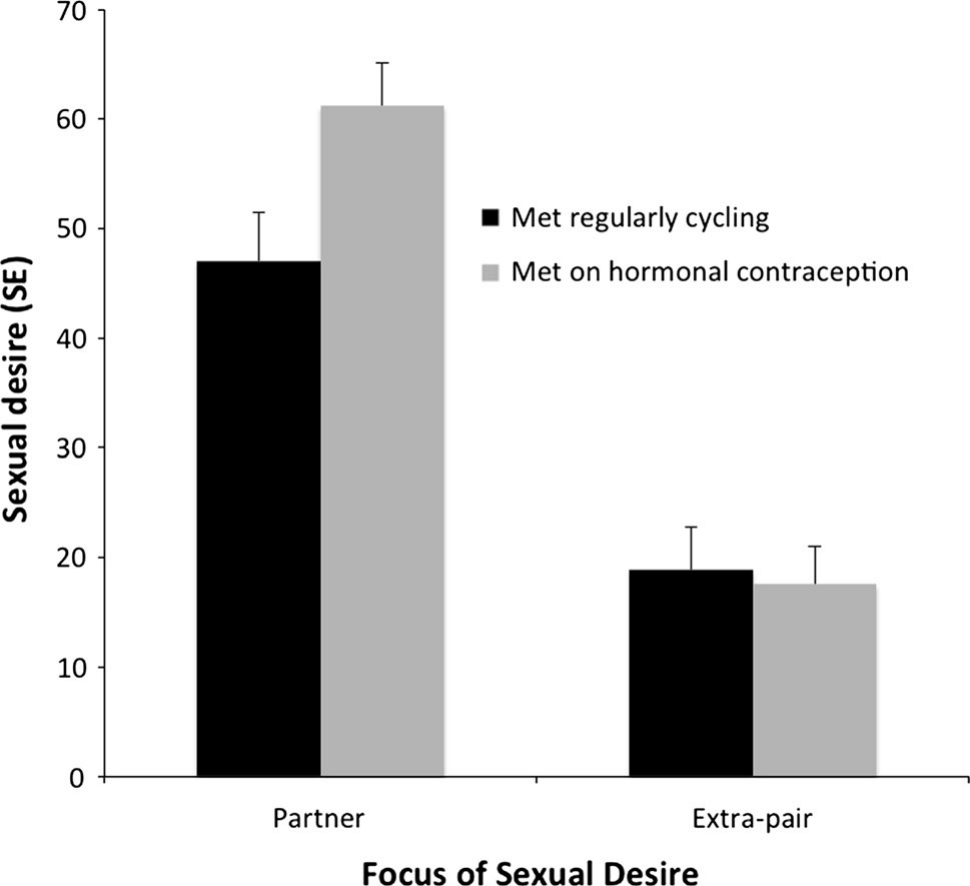
Levels of sexual desire toward the partner and toward extra-pair men among pregnant women who met their partner while regularly cycling (*black*) or while using hormonal contraception (*gray*). Partner-focused sexual desire differed significantly between the two HC groups

We conducted two univariate ANOVAs to examine the impact of women’s use/non-use of hormonal contraception at the time of relationship formation on subsequent levels of sexual desire during pregnancy. We first examined the impact of women’s use/non-use of hormonal contraception on partner-specific sexual desire. We entered scores on the partner-specific sexual desire item as the dependent variable and entered contraceptive use status at the time of mate choice as the fixed-factor. We found that women who met their partner while using hormonal contraception had significantly higher levels of partner-specific sexual desire (*M* = 61.19, *SE* = 3.92) than those who had met when regularly cycling (*M* = 47.05, *SE* = 4.42) (*F*[1, 82] = 5.74, *p* = .02, partial eta squared = .07; see Fig. 1). We then individually added participant age, number of children, education level (5 groups; *N* = 1 missing data), relationship length (*N* = 1 missing data), and gestation stage (in weeks, *N* = 1 missing data) to the ANOVA as covariates to control for these factors. These additions did not change the overall pattern of results, and none of these variables had a significant main effect on the model (all *F* < 2.88, all *p* > .09). To control for potential differences between various formulations of hormonal contraception, we subsequently restricted our analysis to women who indicated that they were using a combined oral contraceptive pill at the time of mate choice (*N* = 31). We then repeated the above analyses and results, though marginal due to reduced power, were similar (*F*[1, 66] = 3.62, *p* = .06).

We then examined the impact of women’s use/non-use of hormonal contraception on levels of extra-pair interest during pregnancy via a second univariate ANOVA. We entered scores on the extra-pair sexual desire item as the dependent variable and again entered contraceptive use status as the fixed-factor. Levels of desire for extra-pair men did not differ among those women who met their partner while regularly cycling (*M* = 18.89, *SE* = 3.90) and those who met their partner while using hormonal contraception (*M* = 17.55, *SE* = 3.46), *F*(1, 82) = .07, *p* = .80. In spite of the fact that ANOVA is robust to violations of normality (Stonehouse & Forrester, 1998; Subrahmaniam, Subrahmaniam, & Messeri, 1975), we conducted a further analysis using a non-parametric tests since normality was violated in the case of the extra-pair desire variable and transformations were unsuccessful. However, results of a non-parametric Mann–Whitney test showed a similar pattern.

## Discussion

Our study is the first to examine sexual desire during pregnancy in relation to hormonal contraceptive use/non-use at the initiation of the relationship. As predicted, we found that women who met their partner while using hormonal contraception had significantly higher levels of sexual desire toward their partner during pregnancy. This result was robust to factors including women’s education level, age, relationship length, and gestation stage, all of which have previously been shown to relate to sexual desire and function (DeLamater & Sill, 2005; Güleroğlu & Beşer, 2014; Hällström & Samuelsson, 1991; Sagiv-Reiss, Birnbaum, & Safir, 2012). We speculate that these findings reflect similarity between women’s partner preferences during pregnancy and during contraceptive pill use; preferences that are distinct from preferences displayed by regularly cycling women (Jones et al., 2005). As predicted, we also found that contraceptive use at the time of relationship initiation did not relate to levels of sexual desire for extra-pair men during pregnancy.

These findings contribute to a growing number of recent studies examining changes in sexual desire and behavior during pregnancy. Since pregnancy is a time of both physical and psychological transition, and is impacted by cultural and social factors, we acknowledge that there is a broad range of variables that are likely to impact sexual desire beyond contraceptive use history. Previous research in this area has, for example, examined factors including women’s body image (Pauls, Occhino, & Dryfhout, 2008) and fear of sexual intercourse in pregnancy (Babazadeh, Najmabadi, Mirzaii, & Masomi, 2013), showing that these factors can be important contributors to women’s sexual desire and function during pregnancy. Nonetheless, our results provide the first evidence that contraceptive use status at the initiation of the relationship explains some variance in partner-specific sexual desire during pregnancy.

Our findings are in line with the predictions of the congruency hypothesis and support the broader proposal that hormonal processes across the female lifespan may influence partner preferences and aspects of relationship functioning including sexual desire. Truly comprehensive research addressing the precise hormonal mechanism responsible for maintaining partner preferences is absent from the literature. Future work assaying multiple hormones and testing women’s mating preferences across the lifespan is thus still needed. We are hesitant to speculate what specific commonalities in the hormonal profile in pregnancy and during hormonal contraceptive use may generate similar partner preferences, which we believe underpin the described effect. Previous research has suggested progesterone may be important for the maintenance of these preferences (Jones et al., 2005; Puts, 2006), yet until research is conducted which assays a broader range of hormones and considers the impact of both exogenous and endogenous hormones, conclusions on this topic are premature. However, if the results were to be explained by a single hormone, such as estrogen or progesterone, we would have expected gestation stage to significantly influence our results. Importantly, considering absolute levels of hormones may be a crude method to identify the hormonal mechanism(s) that maintain women’s mating preferences. As studies in this area are truly in their infancy, it will be critical for future cross-disciplinary work to examine neural mechanisms (e.g., rate change in hormones and binding receptors) to fully understand these processes. Finally, our study did not consider what type of hormonal contraceptive women used when they met their partner. It may be that different brands or concentrations impact mate preferences and behavior differently (e.g., Cobey, Pollet, Roberts, & Buunk, 2011), leading to variation in the levels of congruency in mate preferences during pregnancy. This is something that could be considered in future work, though we doubt the subtle formulation differences among various forms of combined oral contraceptives result in any substantial variation for relationships given the large range of other factors that may influence sexual desire.

A limitation of our work that should be considered is the lack of measurement of actual preferences. As a result, the mechanism for our effect remains undocumented. Though logistically challenging, longitudinal research that assesses women’s mating preferences and hormone levels at the start of their relationship and then during subsequent pregnancy would be an obvious follow-up to the current research. Similarly, future research could improve on our findings by conducting repeated measures of in-pair/extra-pair sexual desire throughout women’s pregnancy and by including corresponding standardized measures of general relationship satisfaction. Specifically, research could address whether or not the effect of congruency is specific to sexual aspects of the relationship or if it has corresponding effects on general satisfaction (e.g., Roberts et al., 2012). Since we used single-item questions to assay partner and extra-pair desire, future research could also strive to replicate our findings using a validated scale. However, to our knowledge, a validated scale, which assays both partner-specific and extra-pair sexual desire, is not currently available (Roberts et al., 2014a). The development of a validated scale that distinguishes between these types of sexual desire would thus be a useful tool for researchers in this area.

Since our study only examined the female partner’s self-reported sexual desire, future research could also build on these findings by examining the male partner’s self-reported sexual desire. Based on the lack of influence of women’s contraceptive congruency on male sexual satisfaction among non-pregnant women (Roberts et al., 2014b), we would predict that there would be no effect of congruency on male sexual desire during pregnancy. However, male partners could further report on their perceptions of their female partner’s sexual desire toward them and toward extra-pair men. This sort of measure would help speak to whether or not differences in women’s sexual desire during pregnancy, as a consequence of contraceptive use at the initiation of the relationship, actually have an impact on relationship functioning or overall satisfaction. As it stands, we cannot be certain that differences in women’s desire actually relate to differences in levels of relationship quality or impact relationship functioning. The current work does not speak to what, if any, reproductive benefits sexual desire during pregnancy may have for couples. We also did not include variables that assayed typical physical symptoms (e.g., sickness, nausea, fatigue) associated with pregnancy or attitudes toward sex during pregnancy. Future research including these variables, and a larger sample size to ensure a representative sample, is thus also warranted.

In sum, these results suggest that hormonal contraceptive use/non-use at the start of the relationship has potential implications for subsequent partner-specific sexual desire during pregnancy. More broadly, our results speak to the need for a broader range of controlled studies examining the impact of hormonal contraceptive use on interpersonal psychological variables (Cobey & Buunk, 2012). Given that pregnancy is a time of great transition, knowledge of factors which affect relationship quality at this time is of considerable value. We certainly are not advocating for or against hormonal contraceptive use based on these findings; naturally, the decision to use/not use hormonal contraception is a complex choice that is a personal decision involving the relative weighting of several different variables of individually relative importance. One important consideration is what impact knowledge of potential changes in sexual desire and function during pregnancy could have for a couple. We suspect that the self-reported changes documented herein, if they indeed have actual consequences for behavior and relationships, ultimately have a relatively minor influence. It may be that awareness of possible changes in levels of sexual desire during pregnancy is sufficient to thwart any negative effects, if these exist (e.g., Serati et al., 2010). We hope this research stimulates further work in this area and prompts the consideration of the broader hormonal mechanisms that underpin women’s mating preferences across the lifespan (Havlíček, Cobey, Barrett, Klapilová, & Roberts, 2015).
